# 4-Dimethyl­amino-1-(4-meth­oxy­phen­yl)-2,5-dioxo-2,5-dihydro-1*H*-pyrrole-3-carbonitrile

**DOI:** 10.1107/S1600536813004893

**Published:** 2013-02-23

**Authors:** Bakr F. Abdel-Wahab, Hanan A. Mohamed, Abdelbasset A. Farahat, Seik Weng Ng, Edward R. T. Tiekink

**Affiliations:** a Applied Organic Chemistry Department, National Research Centre, Dokki, 12622 Giza, Egypt; b Department of Pharmaceutical Organic Chemistry, Faculty of Pharmacy, Mansoura University, Mansoura 35516, Egypt; c Department of Chemistry, University of Malaya, 50603 Kuala Lumpur, Malaysia; d Chemistry Department, Faculty of Science, King Abdulaziz University, PO Box 80203 Jeddah, Saudi Arabia

## Abstract

In the title compound, C_14_H_13_N_3_O_3_, a twist occurs, as seen in the dihedral angle of 53.60 (12)° between the pyrrole and benzene rings. A three-dimensional architecture is formed in the crystal whereby layers of mol­ecules in the *ac* plane are connected by C—H⋯O and C—H⋯π inter­actions.

## Related literature

For background to the biological activity exhibited by pyrroles and pyran­opyrroles, see: Amer *et al.* (2008 ▶, 2009 ▶).
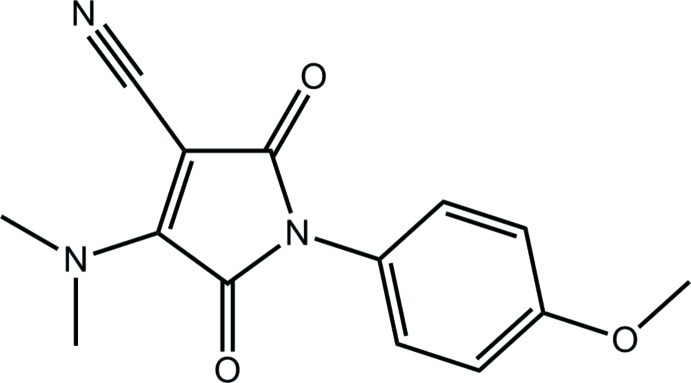


## Experimental

### 

#### Crystal data

C_14_H_13_N_3_O_3_
*M* *_r_* = 271.27
Monoclinic, 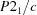

*a* = 12.7408 (14) Å
*b* = 7.8520 (9) Å
*c* = 14.4194 (18) Å
β = 115.163 (14)°
*V* = 1305.6 (3) Å^3^
*Z* = 4
Mo *K*α radiation
μ = 0.10 mm^−1^
*T* = 295 K
0.40 × 0.20 × 0.10 mm

#### Data collection

Agilent SuperNova Dual diffractometer with an Atlas detector
Absorption correction: multi-scan (*CrysAlis PRO*; Agilent, 2011 ▶) *T*_min_ = 0.869, *T*_max_ = 1.000
8113 measured reflections
3020 independent reflections
1772 reflections with *I* > 2σ(*I*)
*R*_int_ = 0.040

#### Refinement

*R*[*F* ^2^ > 2σ(*F* ^2^)] = 0.054
*wR*(*F* ^2^) = 0.153
*S* = 1.04
3020 reflections
184 parameters
H-atom parameters constrained
Δρ_max_ = 0.20 e Å^−3^
Δρ_min_ = −0.17 e Å^−3^

### 

Data collection: *CrysAlis PRO* (Agilent, 2011 ▶); cell refinement: *CrysAlis PRO*; data reduction: *CrysAlis PRO*; program(s) used to solve structure: *SHELXS97* (Sheldrick, 2008 ▶); program(s) used to refine structure: *SHELXL97* (Sheldrick, 2008 ▶); molecular graphics: *ORTEP-3 for Windows* (Farrugia, 2012 ▶) and *DIAMOND* (Brandenburg, 2006 ▶); software used to prepare material for publication: *publCIF* (Westrip, 2010 ▶).

## Supplementary Material

Crystal structure: contains datablock(s) global, I. DOI: 10.1107/S1600536813004893/hb7042sup1.cif

Structure factors: contains datablock(s) I. DOI: 10.1107/S1600536813004893/hb7042Isup2.hkl

Supplementary material file. DOI: 10.1107/S1600536813004893/hb7042Isup3.cml

Additional supplementary materials:  crystallographic information; 3D view; checkCIF report

## Figures and Tables

**Table 1:** Hydrogen-bond geometry (Å, °) *Cg*1 is the centroid of the C8–C13 benzene ring.

*D*—H⋯*A*	*D*—H	H⋯*A*	*D*⋯*A*	*D*—H⋯*A*
C6—H6*A*⋯O2^i^	0.96	2.54	3.397 (3)	149
C12—H12⋯O1^ii^	0.93	2.54	3.384 (3)	151
C5—H5*B*⋯*Cg*1^iii^	0.96	2.94	3.848 (3)	158
C6—H6*B*⋯*Cg*1^iv^	0.96	3.00	3.781 (3)	140

Symmetry codes: (i) 
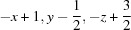
; (ii) 
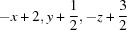
; (iii) 
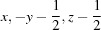
; (iv) 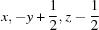.

**Fig. 1.**
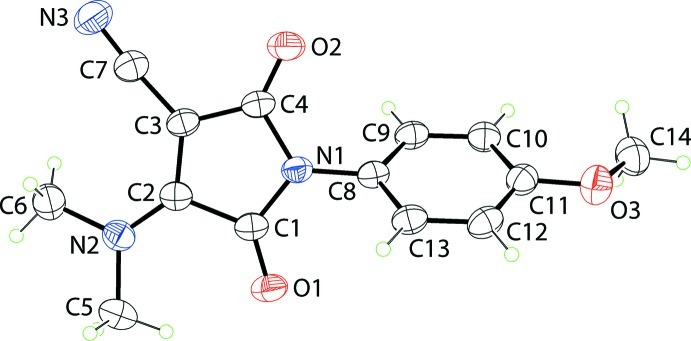
The molecular structure of (I) showing displacement ellipsoids at the 35% probability level.

**Fig. 2.**
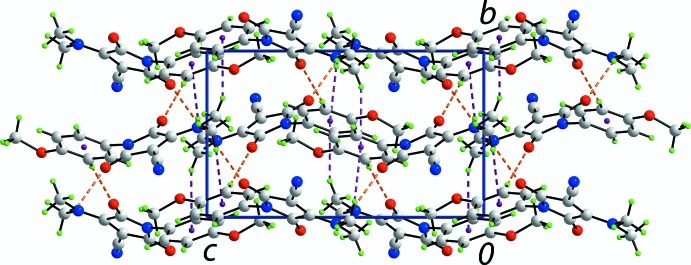
A view of the crystal packing in projection down the a axis. The C—H···O and C—H···π interactions are shown as orange and purple dashed lines, respectively.

## supplementary crystallographic information

### Comment

The title compound (I) was investigated owing to the biological activities exhibited pyrroles and pyranopyrrole analogues (Amer *et al.* 2009; Amer *et al.* 2008). Herein, its crystal structure determination is described.

Crystallography shows that fusion of 1-(4-methoxyphenyl)-4-oxopyrrolidine-3-carbonitrile with excess 1,1-dimethoxy-*N*,*N*-dimethylmethanamine afforded 4-(dimethylamino)-1-(4-methoxyphenyl)-2,5-dioxo-2,5-dihydro-1*H*-pyrrole-3-carbonitrile (I) not the expected 2-((dimethylamino)methylene)-1-(4-methoxyphenyl)-4-oxopyrrolidine-3-carbonitrile (II).

In (I), Fig. 1, the dihedral angle of 53.60 (12)° between the pyrrole (r.m.s. deviation = 0.005 Å) and benzene rings indicates a significant twist in the molecule. The methoxy substituent is twisted out of the plane of the ring to which it is attached as seen in the value of the C14—O3—C11—C10 torsion angle of -13.9 (4)°. The dimethylamino group is also slightly twisted out of the plane through the pyrrole ring to which it is attached; the C5—N2—C2—C1 torsion angle is 8.7 (3)°.

The three-dimensional architecture of (I) is consolidated by C—H···O interactions, involving both carbonyl-O atoms, as well as C—H···π interactions whereby the benzene ring serves as a bridge between molecules, Fig. 2 and Table 1.

### Experimental

A mixture of 1-(4-methoxyphenyl)-4-oxopyrrolidine-3-carbonitrile (0.22 g, 0.001 *M*) and excess 1,1-dimethoxy-*N*,*N*-dimethylmethanamine (0.2 ml) was heated under reflux for about 1.5 h on water bath. The resultant solid was filtered and dried. Re-crystallization was by slow evaporation of its DMF solution which yielded yellow prisms in 28% yield. *M*.pt. 482–483 K.

### Refinement

Carbon-bound H-atoms were placed in calculated positions (C—H 0.93 to 0.96 Å) and were included in the refinement in the riding model approximation, with *U*_iso_(H) = 1.2*U*_equiv_(C).

### Crystal data

**Table:**

C_14_H_13_N_3_O_3_	*F*(000) = 568
*M**_r_* = 271.27	*D*_x_ = 1.380 Mg m^−^^3^
Monoclinic, *P*2_1_/*c*	Mo *K*α radiation, λ = 0.71073 Å
Hall symbol: -P 2ybc	Cell parameters from 1717 reflections
*a* = 12.7408 (14) Å	θ = 2.9–27.5°
*b* = 7.8520 (9) Å	µ = 0.10 mm^−^^1^
*c* = 14.4194 (18) Å	*T* = 295 K
β = 115.163 (14)°	Prism, yellow
*V* = 1305.6 (3) Å^3^	0.40 × 0.20 × 0.10 mm
*Z* = 4	

### Data collection

**Table:**

Agilent SuperNova Dual diffractometer with an Atlas detector	3020 independent reflections
Radiation source: SuperNova (Mo) X-ray Source	1772 reflections with *I* > 2σ(*I*)
Mirror monochromator	*R*_int_ = 0.040
Detector resolution: 10.4041 pixels mm^-1^	θ_max_ = 27.6°, θ_min_ = 2.9°
ω scan	*h* = −16→16
Absorption correction: multi-scan (*CrysAlis PRO*; Agilent, 2011)	*k* = −9→10
*T*_min_ = 0.869, *T*_max_ = 1.000	*l* = −17→18
8113 measured reflections	

### Refinement

**Table:**

Refinement on *F*^2^	Secondary atom site location: difference Fourier map
Least-squares matrix: full	Hydrogen site location: inferred from neighbouring sites
*R*[*F*^2^ > 2σ(*F*^2^)] = 0.054	H-atom parameters constrained
*wR*(*F*^2^) = 0.153	*w* = 1/[σ^2^(*F*_o_^2^) + (0.0573*P*)^2^ + 0.268*P*] where *P* = (*F*_o_^2^ + 2*F*_c_^2^)/3
*S* = 1.04	(Δ/σ)_max_ = 0.001
3020 reflections	Δρ_max_ = 0.20 e Å^−^^3^
184 parameters	Δρ_min_ = −0.17 e Å^−^^3^
0 restraints	Extinction correction: *SHELXL97* (Sheldrick, 2008), Fc^*^=kFc[1+0.001xFc^2^λ^3^/sin(2θ)]^-1/4^
Primary atom site location: structure-invariant direct methods	Extinction coefficient: 0.0065 (17)

### Special details

**Table:**

Geometry. All e.s.d.'s (except the e.s.d. in the dihedral angle between two l.s. planes) are estimated using the full covariance matrix. The cell e.s.d.'s are taken into account individually in the estimation of e.s.d.'s in distances, angles and torsion angles; correlations between e.s.d.'s in cell parameters are only used when they are defined by crystal symmetry. An approximate (isotropic) treatment of cell e.s.d.'s is used for estimating e.s.d.'s involving l.s. planes.
Refinement. Refinement of *F*^2^ against ALL reflections. The weighted *R*-factor *wR* and goodness of fit *S* are based on *F*^2^, conventional *R*-factors *R* are based on *F*, with *F* set to zero for negative *F*^2^. The threshold expression of *F*^2^ > σ(*F*^2^) is used only for calculating *R*-factors(gt) *etc*. and is not relevant to the choice of reflections for refinement. *R*-factors based on *F*^2^ are statistically about twice as large as those based on *F*, and *R*- factors based on ALL data will be even larger.

### Fractional atomic coordinates and isotropic or equivalent isotropic displacement parameters (Å^2^)

**Table:**

	*x*	*y*	*z*	*U*_iso_*/*U*_eq_	
O1	0.87550 (12)	0.4211 (2)	0.83764 (12)	0.0638 (5)	
O2	0.54480 (12)	0.7013 (2)	0.63309 (13)	0.0643 (5)	
O3	0.86456 (14)	0.6113 (3)	0.40226 (13)	0.0700 (5)	
N1	0.72098 (13)	0.5628 (2)	0.71557 (13)	0.0457 (5)	
N2	0.74285 (15)	0.4605 (3)	0.96511 (14)	0.0517 (5)	
N3	0.43202 (18)	0.7134 (3)	0.82506 (18)	0.0764 (7)	
C1	0.78270 (17)	0.4912 (3)	0.81026 (17)	0.0461 (5)	
C2	0.71002 (16)	0.5147 (3)	0.87029 (16)	0.0448 (5)	
C3	0.60974 (16)	0.5976 (3)	0.80526 (16)	0.0463 (6)	
C4	0.61362 (16)	0.6288 (3)	0.70794 (17)	0.0471 (6)	
C5	0.85888 (19)	0.3921 (4)	1.03016 (18)	0.0665 (8)	
H5A	0.9024	0.3797	0.9901	0.100*	
H5B	0.8511	0.2831	1.0567	0.100*	
H5C	0.8987	0.4689	1.0859	0.100*	
C6	0.6647 (2)	0.4773 (4)	1.01516 (18)	0.0644 (7)	
H6A	0.5885	0.4397	0.9693	0.097*	
H6B	0.6615	0.5944	1.0329	0.097*	
H6C	0.6929	0.4089	1.0761	0.097*	
C7	0.51255 (19)	0.6588 (3)	0.81992 (18)	0.0551 (6)	
C8	0.75727 (16)	0.5680 (3)	0.63493 (16)	0.0445 (5)	
C9	0.68625 (17)	0.5068 (3)	0.53895 (17)	0.0501 (6)	
H9	0.6157	0.4570	0.5278	0.060*	
C10	0.71847 (18)	0.5185 (3)	0.45933 (18)	0.0528 (6)	
H10	0.6694	0.4782	0.3947	0.063*	
C11	0.82415 (18)	0.5903 (3)	0.47577 (18)	0.0522 (6)	
C12	0.89700 (18)	0.6489 (3)	0.57264 (18)	0.0558 (6)	
H12	0.9687	0.6952	0.5843	0.067*	
C13	0.86383 (17)	0.6389 (3)	0.65152 (17)	0.0518 (6)	
H13	0.9127	0.6796	0.7161	0.062*	
C14	0.8062 (2)	0.5219 (4)	0.3082 (2)	0.0729 (8)	
H14A	0.8420	0.5479	0.2632	0.109*	
H14B	0.7263	0.5559	0.2766	0.109*	
H14C	0.8111	0.4016	0.3213	0.109*	

### Atomic displacement parameters (Å^2^)

**Table:**

	*U*^11^	*U*^22^	*U*^33^	*U*^12^	*U*^13^	*U*^23^
O1	0.0468 (9)	0.0708 (13)	0.0703 (11)	0.0171 (8)	0.0215 (8)	0.0120 (9)
O2	0.0497 (9)	0.0694 (13)	0.0663 (10)	0.0130 (8)	0.0174 (8)	0.0112 (10)
O3	0.0740 (11)	0.0761 (14)	0.0704 (11)	−0.0066 (9)	0.0409 (9)	0.0007 (10)
N1	0.0349 (9)	0.0490 (12)	0.0492 (10)	0.0021 (8)	0.0143 (8)	0.0038 (9)
N2	0.0497 (10)	0.0507 (13)	0.0499 (11)	−0.0038 (9)	0.0165 (8)	−0.0009 (9)
N3	0.0643 (13)	0.0814 (19)	0.0946 (17)	0.0171 (12)	0.0445 (12)	0.0116 (14)
C1	0.0391 (11)	0.0387 (13)	0.0550 (13)	−0.0014 (9)	0.0146 (9)	−0.0004 (11)
C2	0.0408 (11)	0.0387 (13)	0.0486 (12)	−0.0057 (9)	0.0131 (9)	−0.0051 (10)
C3	0.0373 (11)	0.0429 (14)	0.0555 (13)	−0.0018 (9)	0.0167 (9)	−0.0026 (11)
C4	0.0358 (11)	0.0446 (14)	0.0558 (13)	0.0010 (9)	0.0145 (9)	0.0002 (11)
C5	0.0574 (14)	0.070 (2)	0.0572 (14)	0.0056 (12)	0.0102 (11)	0.0095 (13)
C6	0.0663 (15)	0.071 (2)	0.0569 (15)	−0.0048 (13)	0.0276 (12)	−0.0023 (13)
C7	0.0495 (13)	0.0514 (16)	0.0641 (15)	0.0005 (11)	0.0240 (11)	0.0017 (12)
C8	0.0389 (11)	0.0400 (13)	0.0520 (13)	0.0016 (9)	0.0168 (9)	0.0016 (10)
C9	0.0388 (11)	0.0452 (14)	0.0610 (14)	−0.0034 (9)	0.0162 (10)	0.0007 (12)
C10	0.0474 (12)	0.0538 (16)	0.0530 (13)	−0.0010 (10)	0.0174 (10)	−0.0009 (12)
C11	0.0524 (13)	0.0473 (15)	0.0604 (14)	0.0034 (10)	0.0273 (11)	0.0045 (12)
C12	0.0428 (12)	0.0532 (16)	0.0731 (16)	−0.0064 (10)	0.0262 (11)	−0.0014 (13)
C13	0.0385 (11)	0.0518 (15)	0.0586 (14)	−0.0045 (10)	0.0144 (10)	−0.0051 (12)
C14	0.0936 (19)	0.065 (2)	0.0674 (17)	0.0095 (15)	0.0411 (15)	0.0042 (15)

### Geometric parameters (Å, º)

**Table:**

O1—C1	1.208 (2)	C5—H5C	0.9600
O2—C4	1.206 (3)	C6—H6A	0.9600
O3—C11	1.371 (3)	C6—H6B	0.9600
O3—C14	1.424 (3)	C6—H6C	0.9600
N1—C1	1.373 (3)	C8—C9	1.378 (3)
N1—C8	1.423 (3)	C8—C13	1.391 (3)
N1—C4	1.422 (3)	C9—C10	1.377 (3)
N2—C2	1.319 (3)	C9—H9	0.9300
N2—C6	1.463 (3)	C10—C11	1.384 (3)
N2—C5	1.475 (3)	C10—H10	0.9300
N3—C7	1.143 (3)	C11—C12	1.386 (3)
C1—C2	1.524 (3)	C12—C13	1.374 (3)
C2—C3	1.384 (3)	C12—H12	0.9300
C3—C7	1.425 (3)	C13—H13	0.9300
C3—C4	1.446 (3)	C14—H14A	0.9600
C5—H5A	0.9600	C14—H14B	0.9600
C5—H5B	0.9600	C14—H14C	0.9600
			
C11—O3—C14	117.5 (2)	N2—C6—H6C	109.5
C1—N1—C8	125.20 (17)	H6A—C6—H6C	109.5
C1—N1—C4	110.58 (18)	H6B—C6—H6C	109.5
C8—N1—C4	124.21 (17)	N3—C7—C3	175.1 (3)
C2—N2—C6	119.99 (19)	C9—C8—C13	119.2 (2)
C2—N2—C5	124.6 (2)	C9—C8—N1	120.52 (18)
C6—N2—C5	115.32 (19)	C13—C8—N1	120.23 (19)
O1—C1—N1	125.4 (2)	C10—C9—C8	120.9 (2)
O1—C1—C2	128.0 (2)	C10—C9—H9	119.6
N1—C1—C2	106.60 (17)	C8—C9—H9	119.6
N2—C2—C3	130.4 (2)	C9—C10—C11	119.8 (2)
N2—C2—C1	123.25 (19)	C9—C10—H10	120.1
C3—C2—C1	106.32 (19)	C11—C10—H10	120.1
C2—C3—C7	131.9 (2)	O3—C11—C12	115.4 (2)
C2—C3—C4	109.54 (18)	O3—C11—C10	125.0 (2)
C7—C3—C4	118.50 (18)	C12—C11—C10	119.6 (2)
O2—C4—N1	123.4 (2)	C13—C12—C11	120.4 (2)
O2—C4—C3	129.56 (19)	C13—C12—H12	119.8
N1—C4—C3	106.97 (17)	C11—C12—H12	119.8
N2—C5—H5A	109.5	C12—C13—C8	120.1 (2)
N2—C5—H5B	109.5	C12—C13—H13	119.9
H5A—C5—H5B	109.5	C8—C13—H13	119.9
N2—C5—H5C	109.5	O3—C14—H14A	109.5
H5A—C5—H5C	109.5	O3—C14—H14B	109.5
H5B—C5—H5C	109.5	H14A—C14—H14B	109.5
N2—C6—H6A	109.5	O3—C14—H14C	109.5
N2—C6—H6B	109.5	H14A—C14—H14C	109.5
H6A—C6—H6B	109.5	H14B—C14—H14C	109.5
			
C8—N1—C1—O1	−0.9 (4)	C2—C3—C4—O2	−177.8 (2)
C4—N1—C1—O1	177.9 (2)	C7—C3—C4—O2	−0.2 (4)
C8—N1—C1—C2	−179.66 (19)	C2—C3—C4—N1	−0.4 (3)
C4—N1—C1—C2	−0.9 (2)	C7—C3—C4—N1	177.2 (2)
C6—N2—C2—C3	3.3 (4)	C1—N1—C8—C9	126.3 (2)
C5—N2—C2—C3	−172.2 (2)	C4—N1—C8—C9	−52.3 (3)
C6—N2—C2—C1	−175.7 (2)	C1—N1—C8—C13	−55.3 (3)
C5—N2—C2—C1	8.7 (3)	C4—N1—C8—C13	126.1 (2)
O1—C1—C2—N2	1.2 (4)	C13—C8—C9—C10	−1.4 (4)
N1—C1—C2—N2	179.9 (2)	N1—C8—C9—C10	177.0 (2)
O1—C1—C2—C3	−178.1 (2)	C8—C9—C10—C11	0.9 (4)
N1—C1—C2—C3	0.6 (2)	C14—O3—C11—C12	166.8 (2)
N2—C2—C3—C7	3.6 (4)	C14—O3—C11—C10	−13.9 (4)
C1—C2—C3—C7	−177.2 (2)	C9—C10—C11—O3	−178.9 (2)
N2—C2—C3—C4	−179.3 (2)	C9—C10—C11—C12	0.4 (4)
C1—C2—C3—C4	−0.1 (2)	O3—C11—C12—C13	178.1 (2)
C1—N1—C4—O2	178.4 (2)	C10—C11—C12—C13	−1.3 (4)
C8—N1—C4—O2	−2.8 (4)	C11—C12—C13—C8	0.8 (4)
C1—N1—C4—C3	0.8 (2)	C9—C8—C13—C12	0.6 (4)
C8—N1—C4—C3	179.62 (19)	N1—C8—C13—C12	−177.8 (2)

### Hydrogen-bond geometry (Å, º)

Cg1 is the centroid of the C8–C13 benzene ring.

**Table:**

*D*—H···*A*	*D*—H	H···*A*	*D*···*A*	*D*—H···*A*
C6—H6*A*···O2^i^	0.96	2.54	3.397 (3)	149
C12—H12···O1^ii^	0.93	2.54	3.384 (3)	151
C5—H5*B*···*Cg*1^iii^	0.96	2.94	3.848 (3)	158
C6—H6*B*···*Cg*1^iv^	0.96	3.00	3.781 (3)	140

Symmetry codes: (i) −*x*+1, *y*−1/2, −*z*+3/2; (ii) −*x*+2, *y*+1/2, −*z*+3/2; (iii) *x*, −*y*−1/2, *z*−1/2; (iv) *x*, −*y*+1/2, *z*−1/2.
